# Melt Electrospinning Writing of Magnetic Microrobots

**DOI:** 10.1002/advs.202003177

**Published:** 2021-01-04

**Authors:** Yingchun Su, Tian Qiu, Wen Song, Xiaojun Han, Mengmeng Sun, Zhao Wang, Hui Xie, Mingdong Dong, Menglin Chen

**Affiliations:** ^1^ State Key Laboratory of Urban Water Resource and Environment School of Chemistry and Chemical Engineering Harbin Institute of Technology Harbin 150001 China; ^2^ Interdisciplinary Nanoscience Center (iNANO) Sino‐Danish Center for Education and Research (SDC) Aarhus University Aarhus C DK‐8000 Denmark; ^3^ Department of Engineering Aarhus University Aarhus C DK‐8000 Denmark; ^4^ Max Planck Institute for Intelligent Systems Heisenbergstr. 3 Stuttgart 70569 Germany; ^5^ Institute of Physical Chemistry University of Stuttgart Pfaffenwaldring 55 Stuttgart 70569 Germany; ^6^ State Key Laboratory of Military Stomatology and National Clinical Research Center for Oral Diseases and Shaanxi Key Laboratory of Oral Diseases Department of Prosthodontics School of Stomatology The Fourth Military Medical University Xi'an 710032 China; ^7^ State Key Laboratory of Robotics and Systems Key Laboratory of Microsystems and Microstructures Manufacturing (Ministry of Education) Harbin Institute of Technology Harbin 150080 China

**Keywords:** melt electrospinning writing, micromolding, microrobot, skiving, wireless actuation

## Abstract

The magnetic microrobots actuated by an external magnetic field can access distant, enclosed, and small spaces under fuel‐free conditions, which is apromising technology for manipulation and delivery under microenvironment; however, the complicated fabrication method limits their applications. Herein, three techniques including melt electrospinning writing (MEW), micromolding, and skiving process are combined to successfully mass‐produce tadpole‐like magnetic polycaprolactone/Fe_3_O_4_ (PCL/Fe_3_O_4_) microrobot. Importantly, the tadpole‐like microrobots under an external magnetic field can achieve two locomotions: rolling mode and propulsion mode. The rolling motion can approach the working destination quickly with a speed of ≈2 mm s^−1^. The propulsion motion (0−340 µm s^−1^) can handle a microcargo. Such a simple and cost‐effective production method shows a great potential for scale‐up fabrication of advanced shape‐design, mass‐production, and multifunctionality microrobot.

## Graphical abstract

The tadpole‐like microrobot is controlled wirelessly by an external magnetic field to achieve either propulsion or rolling mode and act as a cargo carrier.

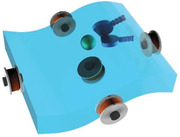

## 1 Introduction

Microrobots were reported with high expectations for inspection and exploration tasks,^[^ ^1^ ^]^ micro‐unmanned air vehicles,^[^ ^2^ ^]^ minimally invasive medical procedures,^[^ ^3^ ^]^ and targeted drug delivery.^[^ ^4^ ^]^ Different types of motors driven by ultrasound,^[^ ^5^ ^]^ light,^[^ ^6^ ^]^ thermal,^[^ ^7^ ^]^ bubbles,^[^ ^8^ ^]^ and magnetic field^[^ ^9^ ^]^ are being continuously developed. Due to the highly controllable magnetic field, magnetically actuated motors have attracted more attention. The up‐to‐date progress on versatile magnetic motors, such as helical motors,^[^ ^10^ ^]^ nanowire motors,^[^ ^11^ ^]^ and colloid motors,^[^ ^12^ ^]^ were reported with controllable movement. Most of them were synthesized with high yields and low‐cost facilities by self‐assembly and electroplating approach.^[^^11b, 12b^^]^ Sperm‐templated method,^[^ ^13^ ^]^ magnetron sputter deposition,^[^ ^14^ ^]^ and self‐scrolling method^[^ ^15^ ^]^ were commonly used approaches for the magnetic motors fabrication. Recently, the tendency of new generation motor requires specific architecture design to achieve desired functions. New strategies for the motor fabrication with designed architectures were developed, such as glancing angle deposition technique^[^ ^16^ ^]^ and 3D laser lithography system.^[^ ^17^ ^]^ Current existing technologies are not accessible to general users. Thus a simple and inexpensive technique is extremely demanded.

Melt electrospinning writing (MEW) technology is an emerging method combining melt electrospinning and 3D printing stage. Compared with the traditional 3D printing, the MEW can realize accurate positioning of submicron fibers with ≈2 orders of magnitude smaller than 3D printing.^[^ ^18^ ^]^ The products as biocompatible scaffolds were mainly applied for cell culture and tissue engineering.^[^ ^19^ ^]^ The MEW realized programmable electrospinning exhibiting high‐level controllability in fibers deposited site, fiber diameter, and fiber shape in a mass‐production way. Through MEW, the complex shapes or geometries can be designed under automated computer control. In scientific studies, microtome is not only an accessory device to produce thin cross‐sections for obtaining optical or electron microscopic images but also has been reported for the fabrication of nano‐ and micromaterials.^[^ ^20^ ^]^ It was proposed as “skiving”^[^ ^21^ ^]^ to get metallic nanowires singly^[^ ^22^ ^]^ or in arrays,^[^ ^23^ ^]^ quantum‐dots,^[^ ^24^ ^]^ and polymeric nanocylinders.^[^ ^25^ ^]^ MEW technology can design microfibers artificially, and skiving technology can section microfibers into thin slices for the fabrication of nano‐ or microstructures.

Herein, the cost‐effective and straightforward MEW method was applied to fabricate magnetic microrobots. MEW directly print the assymetric polycaprolactone (PCL) templates for molding polydimethylsiloxane (PDMS) channels. Subsequently, the channels were filled with PCL/Fe_3_O_4_ mixture. After solidification, the magnetic assymetric billets were demolded, and skived into tadpole‐like magnetic microrobots. The assymetric tadpole‐like magnetic microrobot achieves two independent locomotion (propulsion and rolling) by wireless dynamic magnetic fields. The tadpole‐like microrobot demonstrates well‐controlled manipulation and cargo transportation.

## 2 Results and Discussion

**Figure 1:**
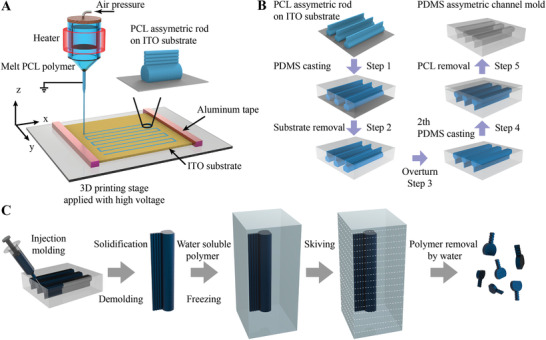
Schematic fabrication process of magnetic tadpole‐like microrobots. Schematic fabrication process of polycaprolactone (PCL) assymetric rod template (A), polydimethylsiloxane (PDMS) assymetric channels (B), and PCL/Fe_3_O_4_ assymetric microrobots (C).

The PCL assymetric template was prepared using a MEW device (**Figure** **1**A). Through conductive aluminum tapes, the moving stage was connected with the conductive slide of an indium tin oxide (ITO) glass substrate, which was printed with the assymetric PCL rods containing a thicker fiber and four identical thinner fibers. After casting with PDMS (Figure 1B, step 1), the ITO glass substrate was removed (Figure 1B, step 2). The PDMS block embedded with assymetric PCL rods was overturned (Figure 1B, step 3) and casted with another PDMS layer (Figure 1B, step 4). To obtain the assymetric channel inside PDMS block, the assymetric PCL template was removed in dichloromethane (DCM) for 3 h (Figure 1B, step 5). By injection molding, the obtained assymetric channels were filled with the magnetic PCL/Fe_3_O_4_ mixture solution (Figure 1C, left image). After solidification, the PCL/Fe_3_O_4_ assymetric billets were demolded and embedded in a frozen water‐soluble polymer, which were skived into microslides with a designated thickness. After removing the water‐soluble polymer, the magnetic tadpole‐like microrobots were obtained (Figure 1C, right image) and ready for further measurements.

**Figure 2:**
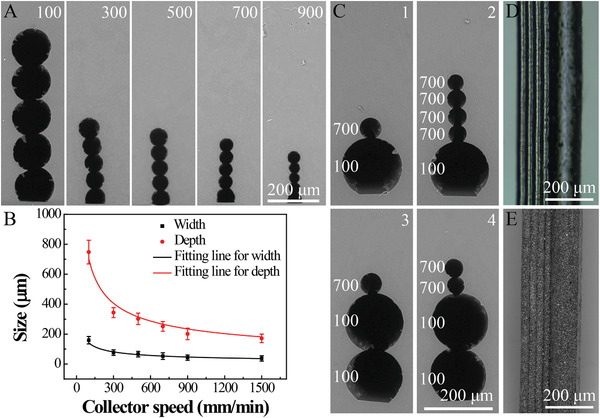
Characterization of the polydimethylsiloxane (PDMS) channels and magnetic polycaprolactone (PCL)/Fe_3_O_4_ assymetric billet. (A) Scanning electron microscopy (SEM) images of the cross‐section of PDMS channels fabricated with different printing speeds using 0.9 mm needles. (B) The width and depth of the channels against the printing speeds. (C) SEM images of the cross‐section of assymetric PDMS channels with designed shapes. (D) Optical microscope image of channel 2. (E) SEM image of magnetic PCL/Fe_3_O_4_ assymetric billet from channel 2. The values of speed were marked on each image and the unit was mm min^−1^. (Mean ± standard deviation (SD) from *n* = 5 different samples fabricated under same condition).

To fabricate tunable PDMS channels with different width and depth, the collector speed was adjusted to obtain PCL rod templates with different diameters and heights. The morphology of PCL fibers at the speed of 100 mm min^−1^ was shown in Figure S1 (Supporting Information). The PDMS channels (**Figure** **2**A) were obtained with five identical layer‐by‐layer PCL fibers at the speed of 100, 300, 500, 700, and 900 mm min^−1^, respectively. By speeding up the collector, the sizes of these PDMS channels were reduced from 158.8 ± 24.8 µm (100 mm min^−1^) to 37.7 ± 17.7 µm (1500 mm min^−1^) for the width and from 748.1 ± 79.0 µm (100 mm min^−1^) to 171.3 ± 28.3 µm (1500 mm min^−1^) for the depth, respectively (Figure 2B). The relationship of depth/width and collector speed was fitted by Equation (1).

(1) $$\begin{array}{c} \mathrm{depth}=5\times \mathrm{width}=5av^{-0.5} \end{array}$$

where *v* and *a* are the collector speed and a constant value, respectively.

The depth of PDMS channels can also be controlled by the number of layers, as shown in Figure S2 (Supporting Information). To prepare assymetric PDMS channels, the speed of the collector for different layers was varied to obtain the assymetric architecture (Figure 2C). The collector speeds for different layers were marked on the left side. For the channel 1 in Figure 2C, the PCL fiber in the first layer was printed at a speed of 100 mm min^−1^, and the collector speed for the second layer was increased to 700 mm min^−1^. The second layer was deposited at the same place as the first layer. The channel 1 contained a thicker fiber and a thinner fiber. While channel 2 contained a thicker fiber and four identical thinner fibers. The channel 3 containing two thicker fibers and a thinner fiber, as well as channel 4 containing two thicker fibers and two thinner fibers were also demonstrated. By using a similar protocol, various other assymetric PDMS channels were also designed (Figure S3, Supporting Information). Importantly, due to the cross‐section of channel 2 possessed the most obvious assymetric structure with a big head and a small tail which is similar to a tadpole, it was chosen for the fabrication of magnetic biomimetic tadpole‐like microrobots in the following section, and optical microscope image of channel 2 was shown in Figure 2D. The scanning electron microscopy (SEM) image of the magnetic PCL/Fe_3_O_4_ assymetric billet from channel 2 can be observed in Figure 2E.

**Figure 3:**
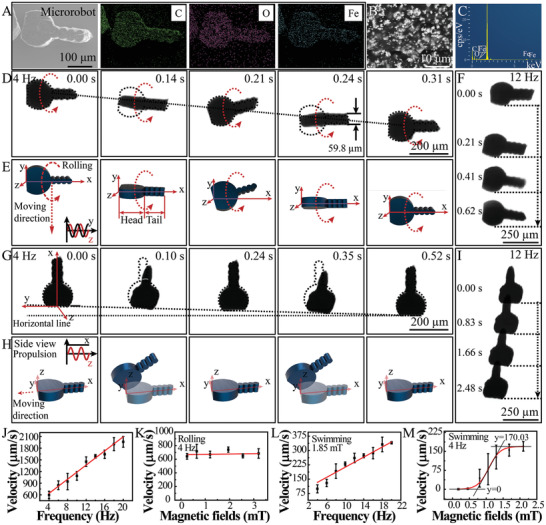
Characterization and controllable movement of the tadpole‐like magnetic microrobot. (A) Scanning electron microscopy (SEM) image of tadpole‐like polycaprolactone (PCL)/Fe_3_O_4_ magnetic microrobot with element mapping of C, O, and Fe. The enlarged SEM image (B) and EDX spectra (C) of the tadpole‐like PCL/Fe_3_O_4_ microrobot. The optical microscope images and 3D schematics of microrobot motion under the rolling (D,E) and propulsion (G,H) magnetic fields (4 Hz, 1.85 mT). The time‐lapse optical images of microrobot motion under the rolling (F) and propulsion (I) magnetic fields (12 Hz, 1.85 mT). Velocities of the tadpole‐like microrobot in the rolling mode (J,K) and the propulsion mode (L,M) against the frequency and intensity (Mean ± SD from *n* = 20 measurements at each experiment).

Inspired by the tadpole‐like morphology, we design a tadpole‐like microrobot using the magnetic PCL/Fe_3_O_4_ assymetric billet translated from channel 2. By skiving the PCL/Fe_3_O_4_ assymetric billet into microslices, the tadpole‐like microrobots were obtained (**Figure** **3**A). The EDX mapping analysis (Figure 3A element distribution images) and EDX spectra (Figure 3C) of the tadpole‐like PCL/Fe_3_O_4_ microrobot prove the coexistence and homogeneous distribution of C, O, and Fe elements. Fe_3_O_4_ clusters were distributed in the magnetic billet (Figure 3B). The elemental analysis indicated 70.58 at% of C, 25.63 at% of O, and 3.79 at% of Fe existence (Table S1, Supporting Information), and by calculating the percentage of C and Fe element, there was about 17.9% (w/w) Fe_3_O_4_ in the magnetic billet.

Our investigation begins with a control experiment to observe line‐shaped motion under a rotating magnetic field with a frequency of 4 Hz and an intensity of 1.85 mT, where the rolling motion of the microrobot on a surface was achieved. In previous reports, the rolling motions of magnetic microrobots applied for cargo transport have been well studied under rotating magnetic field.^[^ ^26^ ^]^ Figure 3D shows the microscopic image sequence of the rolling motion, and Figure 3E demonstrates the corresponding 3D kinematic model of the microrobot. The brief schematic curve of applied magnetic field along *z* axis and *y* axis was shown as the inset of the first image in Figure 3E, resulting in a resultant magnetic field that rotates at a constant frequency in the *y*−*z* plane. When applying a rotating magnetic field around the *x*‐axis, the microrobot rolls along its long axis on the surface and moves along the *y* direction. The front and side appeared sequentially in Figure 3D, proving the continuous rolling motion (see also Movie S1, Supporting Information). Figure 3F captured different time‐stamps for the microrobot under a rotating magnetic field with a frequency of 12 Hz and an intensity of 1.85 mT showing that the microrobot was rolling along a predefined line‐shaped track in 0.62 s. Due to the asymmetric structure of microrobots, a deviation angle of ≈4.2° in the direction of movement can be observed. The tail of the tadpole‐like microrobot was rigid, as the tail of microrobots was tilting together with the head accordingly (Figure 3D,G). According to initial magnetization states, the microrobot can also rotate along the short axis (Movie S2, Supporting Information). For two microrobots, the rolling magnetic field also exhibited reasonable control with the exactly same motion by rolling along the short axis (Movie S3, Supporting Information). The robot switched the rotating directions very fast following the switching of the magnetic field, and it showed a linear increase of the rolling speed (from 598.7 ± 93.8 µm s^−1^ to 2.0 ± 0.1 mm s^−1^) with the rise in the driving frequency up to 20 Hz (Figure 3J), which was recorded by an optical microscope, as shown in Movie S4 (Supporting Information). In Table S2 (Supporting Information), we present a summary of recent magnetic microrobot papers, and our rolling motion exhibited a relatively high velocity. However, when the frequency was fixed at 4 Hz, the intensity of the magnetic field did not significantly affect the rolling velocity (Figure 3K).

Subsequently, a propulsion magnetic field, which comprised a constant magnetic field of 2.5 mT in the *x*−*y* plane and a sinusoidal magnetic field with a swing amplitude between 1.85 and −1.85 mT along *z* axis (Figure S4A and S4B, Supporting Information) was applied. The value of the resultant magnetic field in *x*−*y* plane is constant, the angle between *x*‐axis or *y*‐axis was tunable by the applied magnetic field along the *x*‐axis and *y*‐axis. To simplify the model, the magnetic field along y‐axis was ignored and only a constant magnetic field along *x*‐axis was applied (2.5 mT). When the field intensity of the constant magnetic field along *x*‐axis becomes negative (−2.5 mT), the direction of movement will be turned by 180°. The central axis of microrobot can swing up along the direction of the resultant magnetic field (Figure S4C, Supporting Information), and the theoretical angle *α* between the resultant magnetic field and the constant magnetic field was shown in Figure S4D (Supporting Information). According to the calculation, the maximum angle *α* should be 36.5°. Due to the space constraints by the substrate, the microrobot can only follow the positive angles. The optical images (Figure 3G) and the 3D isometric views (Figure 3H) under a sinusoidal magnetic field of 4 Hz were exhibited to show the propulsion movements. By measuring the length of the shadow at 0.1 s of 227.3 µm, the angle between the central axis of tadpole‐like microrobot and the *x*−*y* plane is 35.7° with the tail moving up. The angle between the central axis of the microrobot and the *x*‐axis is 8.9°, which means in the *x*−*y* plane the microrobot rotated 8.9° clockwise. The complex propulsion movement with tails up and down was due to the complex joint effect of magnetic force, friction, gravity, and fluid mechanics.^[^ ^27^ ^]^ By capturing different time‐stamps for the microrobot under the propulsion magnetic field (12 Hz), a predefined line‐shaped track in 2.48 s was observed (Figure 3I) with a deviation angle of ≈10.3° In the propulsion motion mode, the increasing frequency of magnetic field along *z* axis can speed up the propulsion linearly (Figure 3L), and the changes in velocity (from 99.2 ± 21.2 µm s^−1^ at 4 Hz to 340.4 ± 6.2 µm s^−1^ at 20 Hz) are not as dramatic as that of rolling movement. The propulsion movement of the microrobot under different frequencies from 4 to 20 Hz was shown in Movie S5 (Supporting Information). As for the changes in intensity from 0.18 to 2.1 mT fitted by a nonlinear sigmoidal function, it was noted that only when the strength was larger than a threshold of ≈0.46 mT, the microrobot can be driven. And the velocity increased as the intensity increased and then reached the velocity saturation around 170.03 µm s^−1^.

**Figure 4:**
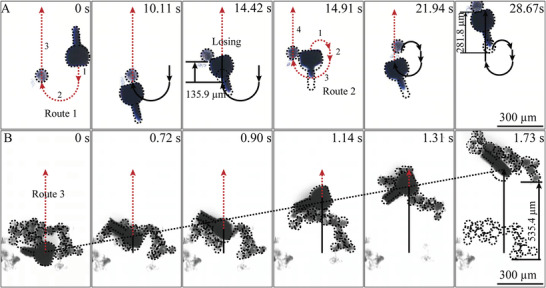
The optical microscope images of the tadpole‐like microrobot carrying microballs under a magnetic field of 4 Hz and 1.85 mT in two motions. (A) The propulsion mode by the head for single cargo manipulation. (B) The rolling mode for multicargos transportation.

Importantly, the possibility of cargo manipulation using the magnetically propelled tadpole‐like microrobot was investigated. The front profile of the tadpole‐like microrobot and the calcium phosphate (CaP) ball‐like cargo was marked by the black dotted line. One cargo manipulation process was shown in **Figure** **4**A (see also Movie S6, Supporting Information). The microrobot propels to approach a spherical cargo (≈89.8 µm in diameter) using its head in the designed route 1, which consists of three sections (1, a straight line; 2, a semicircle; 3, a straight line) and enables the efficient cargo manipulation along the designed motion trajectory. After touching, it moved the ball ahead about 135.9 µm with a speed of 31.5 µm s^−1^. Due to the head of the tadpole‐like microrobot was not parallel with the horizontal line, the ball‐like cargo was lost. After losing the ball, the tadpole‐like microrobot was manually controlled to approach it again using designed route 2. Due to the ball was offset from the specified location, the section 3 was temporarily changed by a smaller semicircular path, as marked in the images captured at 21.94 s. After that, the microrobot moved the cargo ahead again for an extra distance of 281.8 µm from 21.94 s to 28.67 s (41.9 µm s^−1^). In addition to the single cargo manipulation, the rolling microrobot can also achieve multicargos transportation by rolling together in a straight route 3 (Figure 4B and Movie S7, Supporting Information). The multicargos were moved by 535.4 µm with a velocity of 309.5 µm s^−1^. The microrobot fabricated by MEW technology demonstrates controllable motion movement and cargo transportation capability under wireless magnetic control.

## 3 Conclusion

Herein, we successfully produced the magnetic tadpole‐like microrobots achieving a rolling or propulsion movement by controlling magnetic fields. The frequency, intensity, and direction of the magnetic field can conveniently modulate the velocity and direction of the tadpole‐like microrobot. The tadpole‐like microrobot can also transport one cargo or multicargos to a defined destination in a microenvironment with efficient motion and precise controllability. Advances in MEW have enabled the construction of microrobot with designable cross‐section morphology. Based on precise skiving process from billets to microslices, the fabrication process possessed highly repeatable, low‐cost, and mass‐production ability. Importantly, by introducing different functional nanomaterials in molding process, the microrobot can be designed with different functions, or even multifunction to produce bioinspired microrobots. The combination of MEW, micromolding, and skiving technology held great potential to fabricate versatile microrobots.

## 4 Experimental Section

##### Materials

PCL (Mn 45 kD and Mn 80 kD), ITO coated glass slide, DCM, Fe_3_O_4_ nanopowder (50−100 nm particles), chloroform (CF), ethanol, K_2_HPO_4_, and CaI_2_ were purchased from Sigma‐Aldrich (USA). SYLGARD 184 silicone elastomer kit (base and curing agent) was purchased from Dow Corning Company (USA). Tissue‐Tek optimum cutting temperature (O.C.T.) compound was obtained from Sakura Finetechnical (Japan). All chemicals were used without any further purification. Ultrapure water (18.2 MΩ cm) was used for solution preparation in all experiments.

##### Preparation of PCL Assymetric Rod Templates

PCL pellets (Mn 45 kDa, 1 g) were loaded into a Spraybase melt electrospinning instrument (Ireland) and heated to 70 °C for about 1 h to get a homogeneous polymer melt. The tip‐collector distance, the applied voltage, and the pressure were set to be 4.0 mm, 3.5 kV, and 0.25 bar, respectively. ITO coated glass slide (conductive side up) was placed on a flat‐plate stage using conductive aluminum tape. The 20 G (0.9 mm in diameter) stationary flat needle was grounded. The motion of programmable stage was controlled by UCCNC software (using G‐code). By controlling the stage speed, the PCL assymetric rod templates were electrospun on the ITO substrate.

##### Preparation of PDMS Assymetric Channel Mold

PDMS base (7 g) and curing agent (0.7 g) were thoroughly mixed. The air bubbles in the mixture were removed by repeating air extraction using a vacuum desiccator. The mixed solution was poured into a petri dish containing the ITO substrate with printed PCL assymetric rods (conductive side up). After curing overnight, the ITO substrate was removed to leave the PDMS block embedded with PCL assymetric rods. Afterward, it was overturned and coated with another PDMS layer. The prepared PDMS block was cut to expose both ends of the PCL assymetric rods. Followed by soaking in DCM for 3 h, the PCL polymer was removed, and the PDMS assymetric channels were obtained. The morphology of PDMS assymetric channel was characterized by SEM (HITACHI, Tabletop microscope) at an accelerating voltage of 15 kV.

##### Preparation of Magnetic PCL/Fe_3_O_4_ Assymetric Billet

Fe_3_O_4_ nanopowders (0.1 g) were dispersed in 2.5 mL CF under sonication for 1 h. Then 0.5 g of PCL pellets (Mn 80 kDa) was added under magnetic stirring for 24 h. The PCL and Fe_3_O_4_ mixture solution was infused into the empty channel mold by injection syringe with a ≈0.4 mm external diameter needle. The PDMS assymetric channels with PCL/Fe_3_O_4_ mixture solution were volatilized in the fume hood overnight. After solidification, the PDMS channels were carefully opened, and the magnetic PCL/Fe_3_O_4_ assymetric billets were pulled out.

##### Preparation of Magnetic Tadpole‐Like Microrobots

The magnetic PCL/Fe_3_O_4_ assymetric billet was frozen at −80 °C with the Tissue‐Tek O.C.T. compound. The magnetic PCL/Fe_3_O_4_ assymetric billet with O.C.T. compound coating was skived at a thickness of 60 µm using a freezing microtome (Leica CM 1850, Germany) at −20 °C. The morphology of the PCL/Fe_3_O_4_ magnetic tadpole‐like microrobot was characterized by SEM (Quanta 200 FEG; Netherlands).

##### Magnetic Drive Setup

The same magnetic drive setup was used in other literatures.^[^ ^12^, ^28^ ^]^ The schematic configuration of the magnetic drive setup was shown in Figure S5 (Supporting Information). The customized magnetic manipulation system consisted of two pairs of oppositely Helmholtz coils providing independent magnetic forces along the *x*‐axis and *y*‐axis, and a single coil underneath producing the *z*‐axis magnetic force. The magnetic manipulation system was mounted on the dual axis *x*−*y* micropositioner stage with a travel range of 0−15 mm to locate samples. Directly above the magnetic manipulation system, the optical microscope (20×) and charge‐coupled device (CCD) camera were used for the movement recording. A highspeed data acquisition card (NI‐PCI‐6259) provided an in‐put driving signal, which was amplified by the piezoelectric actuator to produce a magnetic field through the magnetic manipulation system. With a drive voltage of 4 V, the strength of the magnetic field could reach 4 mT. Magnetic field control was performed by the multithread software developed on the LabVIEW platform together with a Xiaomi wireless bluetooth joystick.

##### Preparation of CaP Microballs

The CaP microballs were fabricated according to the method described in our previous paper.^[^ ^29^ ^]^ A total of 500 µL of saturated K_2_HPO_4_ aqueous solution was injected into 50 mL ethanol under magnetic stirring to prepare K_2_HPO_4_ crystal clusters. Then 10 mL K_2_HPO_4_ crystals in ethanol and 10 mL 0.7 mol L^−1^ CaI_2_ ethanol solution were mixed well. After adding 20 mL of water, the CaP microballs were produced. The CaP microballs were washed with ultrapure water for three times and stored in ultrapure water.

##### Statistical Analysis

All the lengths and distances were measured by ImageJ software. Experiments to measure the width and depth of the channels (Figure 2) were repeated on five different samples realized with the same fabricating conditions. The velocities of microrobots were calculated by the measured distance divided by time. The 20 different distances were measured, and the corresponding time periods were recorded for each condition.

## Conflict of Interest

The authors declare no conflict of interest.

## Supporting information

Supporting Information

Supplemental Movie 1

Supplemental Movie 2

Supplemental Movie 3

Supplemental Movie 4

Supplemental Movie 5

Supplemental Movie 6

Supplemental Movie 7
