# Supplementary material for: Melt Electrospinning Writing of Magnetic Microrobots
Source: Adv Sci (Weinh). 2021 Jan 4;8(3):2003177. doi: 10.1002/advs.202003177 (PMC7856894; doi:10.1002/advs.202003177)
Supplement: Supplementary file 1 — Supporting Information [file ADVS-8-2003177-s001.pdf]

WILEY-VCH

© 2020 Wiley-VCH GmbH

ADVANCED SCIENCE
Open Access

Supporting Information

for *Adv. Sci.,* DOI: 10.1002/advs.202003177

Melt Electrospinning Writing of Magnetic Microrobots

Yingchun Su, Tian Qiu, Wen Song, Xiaojun Han, Mengmeng Sun, Zhao Wang, Hui Xie, Mingdong Dong,* and Menglin Chen**

© 2020 Wiley-VCH GmbH

Supporting Information

Melt Electrospinning Writing of Magnetic Microrobots

Yingchun Su, Tian Qiu, Wen Song, Xiaojun Han, Mengmeng Sun, Zhao Wang, Hui Xie, Mingdong Dong,* and Menglin Chen**

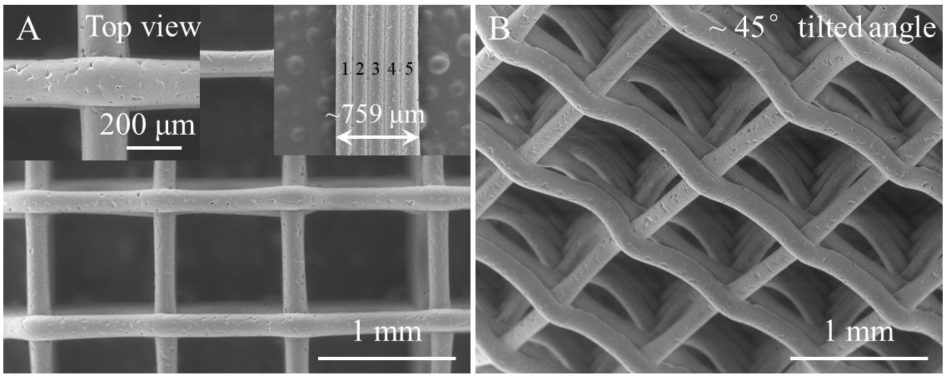

Figure S1. The SEM images of PCL fibers. 0 ° (A) and ~ 45 ° (B) tilted angle with the tip-collector distance of 4 mm, the applied voltage of 3.5 kV, the air pressure of 0.25 bar, the stage speed of 100 mm/min and the needle diameter of 0.90 mm.

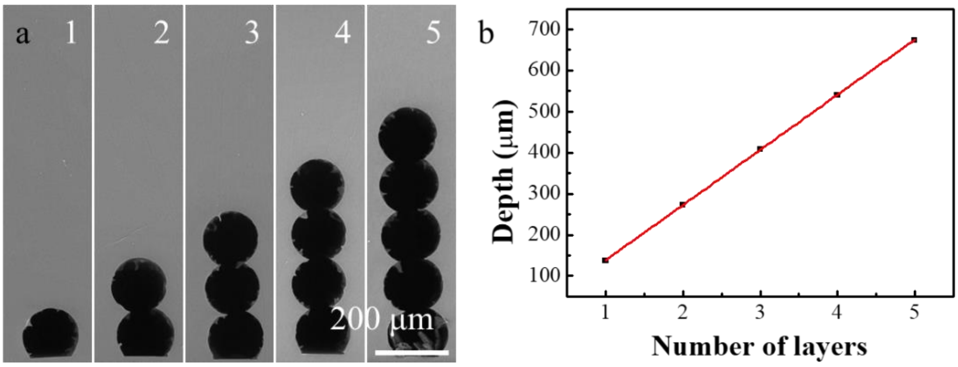

Figure S2. Characterization of PDMS channels made from fibers with different layers. (A) SEM images of PDMS channels made from fibers with different layers. (B) The depth of PDMS channels against different layers and linear fitting curve.

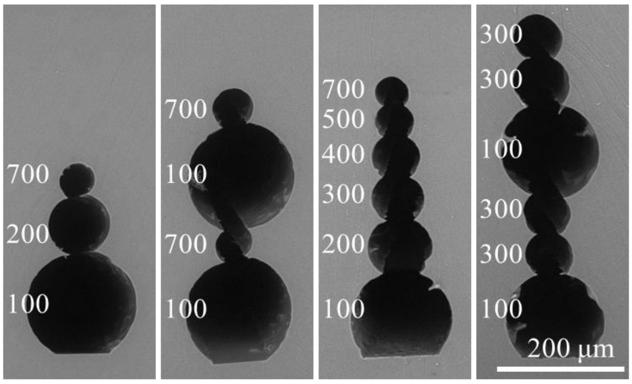

Figure S3. SEM images of different designed PDMS assymetric channels.

Table S1. The elemental analysis of PCL/Fe_3O_4 assymetric magnetic robot.

Elements	wt%	at%
C	57.69	70.58
O	27.90	25.63
Fe	14.40	3.79

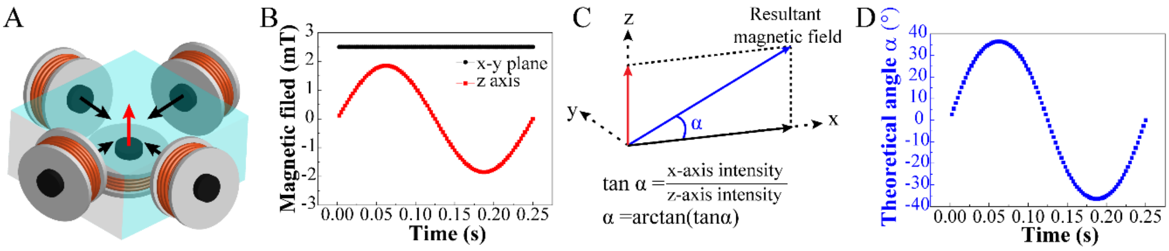

Figure S4. Magnetic manipulation system and corresponding magnetic field. (A) Schematic of magnetic system and corresponding magnetic field; (B) The intensity curve along different time for the component propulsion magnetic field in x-y plane and along z axis; (C) Schematic diagram to calculate the angle α between resultant magnetic field and x axis; (D) The theoretical angle α vs times.

Table S2. A summary of recent literatures about magnetic microrobots.

Type of magnetic robot	Size	Actuation methods	The fastest velocity	Magnetic field	Refs.
Tadpole-like PCL/Fe_3O_4 microrobot	~ 60 μm in thickness and 276 ± 50 μm in length	Rotating magnetic field	2.0±0.1 mm/s	1.85 mT magnetic field with a frequency 20 Hz	This work
		Propulsion magnetic field	340.4±6.2 μm/s		
Burr-like porous spherical microrobot	Diameter ranged from 70 to 90 μm	Field gradient	~1.5 mm/s	20 T/m gradient	[1]
Bilayer hydrogel microsheet robot	80 μm thick and 800-1000 μm long	Field gradient	~1.2 mm/min	20 mT magnetic field and 2 mT/m gradient	[2]
A silicon carbonitride ceramic cylindrical microrobot	42 μm long	Rotating magnetic field	85.56 μm/s	22 mT magnetic field with a frequency 7 Hz	[3]
A soft two-tailed microrobot with a magnetic head and two collinear, unequal, and opposite tails	A 215 μm long first tail; the second tail length ranges of 215 - 430 μm; 25 and 80 μm in minor and major head diameter, respectively	Oscillated field	35.2 μm/s	18 mT magnetic field with a frequency 6 Hz and 5 T/m gradient	[4]
Superparamagnetic microparticle chain	5 μm in diameter	Rotating magnetic field	6.9 ± 2.9 μm/s	<10 mT magnetic field with a frequency 5 Hz; tilt angle of 40° and precession angle of 45°	[5]

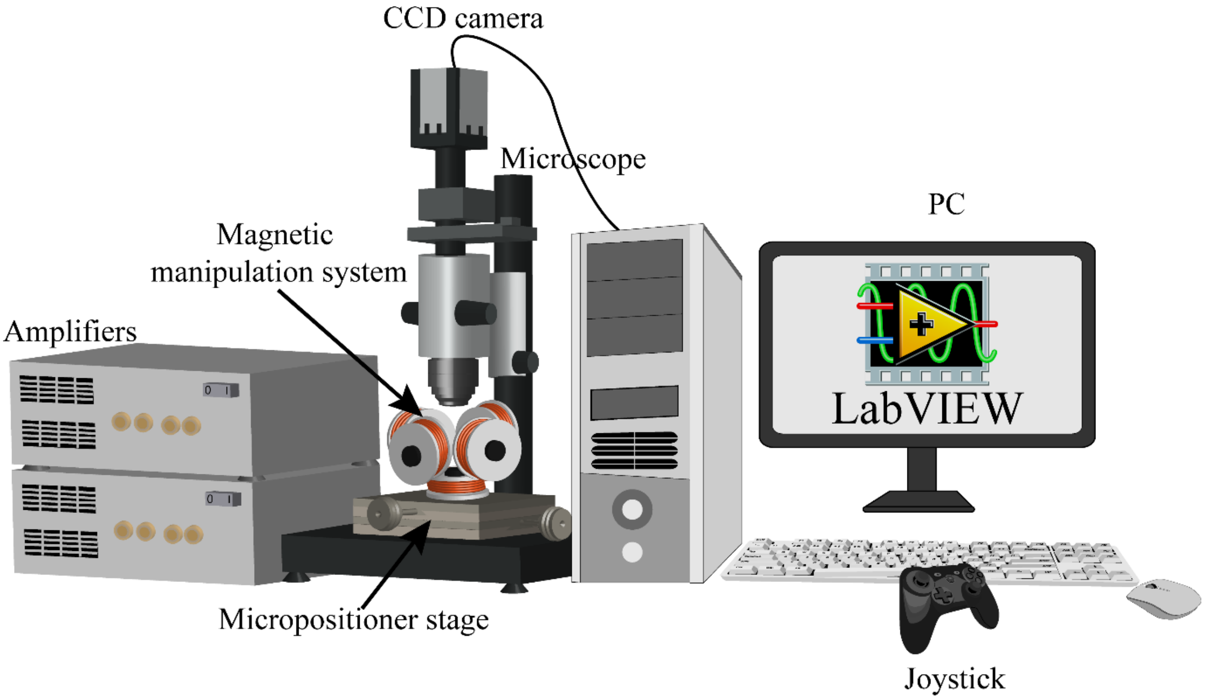

Figure S5. Schematic of magnetic drive setup.

References

[1] J. Y. Li, X. J. Li, T. Luo, R. Wang, C. C. Liu, S. X. Chen, D. F. Li, J. B. Yue, S. H. Cheng, D. Sun, *Sci. Robot.* **2018**, *3*, eaat8829.
[2] D. I. Kim, H. Lee, S. H. Kwon, Y. J. Sung, W. K. Song, S. Park, *Adv. Healthc. Mater.* **2020**, *9*, 2000118.
[3] K. W. Gyak, S. Jeon, L. Ha, S. Kim, J. Y. Kim, K. S. Lee, H. Choi, D.P. Kim, *Adv. Healthc. Mater.* **2019**, *8*, 1900739.
[4] I. S. M. Khalil, A. F. Tabak, Y. Hamed, M. E. Mitwally, M. Tawakol, A. Klingner, M. Sitti, *Adv. Sci.* **2018**, *5*, 700461.
[5] B. Yigit, Y. Alapan, M. Sitti, *Adv. Sci.*, **2019**, *6*, 1801837.
